# Supplementary material for: Identifying the engagement of a brain network during a targeted tDCS-fMRI experiment using a machine learning approach
Source: PLoS Comput Biol. 2023 Apr 12;19(4):e1011012. doi: 10.1371/journal.pcbi.1011012 (PMC10124825; doi:10.1371/journal.pcbi.1011012)
Supplement: S1 Document — A) Information regarding specific settings used in the GRETNA software while extracting the DFCs; B) ROI sets used for analysis and prediction of DFCs of tDCS; C) ROC and MCC across different ROIs for the classification shown by various models with data from only the first 5 DFC matrices is included; D) Accuracy across different ROIs for the classification shown by various models with data from only the first 5 DFC matrices is included; E) ROC and MCC across different ROIs for the classification shown by various models data from only the first 10 DFC matrices is included; F) Accuracy across different ROIs for the classification shown by various models data from only the first 10 DFC matrices is included; G) Top 10 ROI Pairs contributing the classification using the dataset produced by 26 ROIs; and H) Top 10 ROI Pairs contributing the classification when used dataset produced by 22 ROIs. (PDF) [file pcbi.1011012.s001.pdf]

Supplementary Information

Table A presents data on the specific settings or options that were chosen in the process of calculating dynamic functional connectivity (DFC) for each session using the GRETNA software. The GRETNA software is a tool that allows researchers to analyze functional magnetic resonance imaging (fMRI) data and calculate DFC.

Table A: Information regarding specific settings used in the GRETNA software while extracting the DFCs.

**Parameters**	**Specific Input**
Slice Order	Alternating in the plus direction (starting at odd)
Reference Slice	Middle Slice
Normalization Strategy	DARTEL
Voxel Sizes (mm)	[1 1 1]
FWHM Size (mm)	[4 4 4]
Detrending Order	Linear and Quadratic
Regress Out Covariates	White Matter Signal, CSF Signal, Head Motion Strategy: Friston – 24 Parameters
Interpolation Strategy	Nearest Interpolation
Fisher's Z Transformation	True
Sliding Window Step	1
Sliding Window Length	Varies according to TR

Table B represents the ROIs which were identified to evaluate the effects of tDCS.

Table B: ROI sets used for analysis and prediction of DFCs of tDCS.

No.	16 ROIs	22 ROIs	26 ROIs	38 ROIs
1	IFG1_L	IFG1_L	IFG1_L	IFG1_L
2	IFG1_R	IFG1_R	IFG1_R	IFG1_R
3	IFG3_L	IFG3_L	IFG3_L	IFG2_L
4	IFG3_R	IFG3_R	IFG3_R	IFG2_R
5	IFG4_L	IFG4_L	IFG4_L	IFG3_L
6	IFG4_R	IFG4_R	IFG4_R	IFG3_R
7	IFG5_L	IFG5_L	IFG5_L	IFG4_L
8	IFG5_R	IFG5_R	IFG5_R	IFG4_R
9	occMed_L	occMed_L	iPreCG_L	IFG5_L
10	occMed_R	occMed_R	iPreCG_R	IFG5_R
11	SMG1_L	ParOper_L	occMed_L	iPreCG_L
12	SMG1_R	ParOper_R	occMed_R	iPreCG_R
13	SMG3_L	SMA1_L	ParOper_L	mPreCG_R
14	SMG3_R	SMA1_R	ParOper_R	mPreCG_L
15	tempMed_L	SMA2_L	pSTG_L	occLat_L
16	tempMed_R	SMA2_R	pSTG_R	occLat_R
17		SMG1_L	SMA1_L	occMed_L
18		SMG1_R	SMA1_R	occMed_R
19		SMG3_L	SMA2_L	ParOper_L
20		SMG3_R	SMA2_R	ParOper_R
21		tempMed_L	SMG1_L	pMTG_L
22		tempMed_R	SMG1_R	pMTG_R
23			SMG3_L	pSTG_L
24			SMG3_R	pSTG_R
25			tempMed_L	SMA1_L
26			tempMed_R	SMA1_R
27				SMA2_L
28				SMA2_R
29				SMG1_L
30				SMG1_R
31				SMG_L
32				SMG2_R
33				SMG3_L
34				SMG3_R
35				tempLat_L
36				tempLat_R
37				tempMed_L
38				tempMed_R

Tables C and D represent the sub-analyses: data from only the first 5 DFC matrices is included.

Table C: ROC and MCC across different ROIs for the classification shown by various models

Model	No. of ROIs - 112		No. of ROIs - 38		No. of ROIs - 26		No. of ROIs - 22		No. of ROIs - 16	
	ROC	MCC	ROC	MCC	ROC	MCC	ROC	MCC	ROC	MCC
K Neighbors Classifier	**0.517**	**0.478**	0.511	0.486	**0.569**	**0.533**	0.444	0.422	**0.513**	0.451
Random Forest Classifier	0.438	0.422	0.487	0.462	0.532	0.477	0.321	0.291	0.451	0.422
Logistic Regression	0.467	0.443	0.511	0.487	0.542	0.522	0.431	0.397	0.398	0.355
Decision Tree Classifier	0.458	0.421	**0.531**	**0.498**	0.544	0.531	**0.578**	**0.552**	0.512	**0.483**
Naive Bayes	0.358	0.347	0.421	0.396	0.317	0.289	0.346	0.331	0.341	0.321

Table D: Accuracy across different ROIs for the classification shown by various models

Model	No. of ROIs - 112	No. of ROIs - 38	No. of ROIs - 26	No. of ROIs - 22	No. of ROIs - 16
K Neighbors Classifier	**0.541**	**0.522**	**0.612**	0.438	**0.561**
Random Forest Classifier	0.459	0.517	0.577	0.362	0.431
Logistic Regression	0.431	0.468	0.442	0.418	0.351
Decision Tree Classifier	0.437	0.521	0.573	**0.592**	0.485
Naive Bayes	0.328	0.385	0.428	0.366	0.342

Tables E and F represent the sub-analyses when data included is from the first 10 DFC matrices.

Table E: ROC and MCC across different ROIs for the classification shown by various models

Model	No. of ROIs - 112		No. of ROIs - 38		No. of ROIs - 26		No. of ROIs - 22		No. of ROIs - 16	
	ROC	MCC	ROC	MCC	ROC	MCC	ROC	MCC	ROC	MCC
K Neighbors Classifier	0.415	0.387	0.411	0.375	0.525	0.475	0.483	0.468	0.397	0.365
Random Forest Classifier	0.441	0.435	0.475	0.461	0.379	0.388	0.477	0.451	0.308	0.287
Logistic Regression	0.539	0.527	0.531	0.487	0.418	0.387	0.511	0.495	**0.629**	**0.572**
Decision Tree Classifier	0.431	0.429	0.497	0.476	0.455	0.432	0.442	0.403	0.399	0.365
Naive Bayes	**0.574**	**0.535**	**0.543**	**0.526**	**0.599**	**0.532**	**0.555**	**0.522**	0.598	0.549

Table F: Accuracy across different ROIs for the classification shown by various models

Model	No. of ROIs - 112	No. of ROIs - 38	No. of ROIs - 26	No. of ROIs - 22	No. of ROIs - 16
K Neighbors Classifier	0.427	0.487	0.416	0.478	0.375
Random Forest Classifier	0.433	0.477	0.366	0.491	0.326
Logistic Regression	0.512	0.468	0.388	**0.538**	**0.649**
Decision Tree Classifier	0.389	0.528	0.446	0.412	0.384
Naive Bayes	**0.543**	**0.587**	**0.561**	0.536	0.588

Table G represents the ROI pairs contributing highest towards the classification predicted by the algorithms when using 26 ROIs

Table G: Top 10 ROI Pairs contributing the classification using the dataset produced by 26 ROIs

ROI Pair Codes	ROI - 1	ROI - 2
A146	SMA1_R	iPreCG_R
A82	ParOper_R	IFG3_R
A245	SMG3_L	ParOper_R
A244	SMG3_L	ParOper_L
A238	SMG3_L	IFG5_L
A93	pSTG_L	IFG1_R
A216	SMG1_R	IFG4_R
A300	tempMed_L	SMG3_R
A145	SMA1_R	iPreCG_L
A178	SMA2_R	IFG5_L

Table H represents the ROI pairs contributing highest towards the classification predicted by the algorithms when using 22 ROIs

Table H: Top 10 ROI Pairs contributing the classification when used dataset produced by 22 ROIs

ROI Pair Codes	ROI - 1	ROI - 2
A134	SMA1_L	SMG3_R
A161	SMA2_L	IFG5_R
A49	SMG1_L	IFG3_R
A164	SMA2_L	SMG1_L
A118	tempMed_R	SMG3_L
A55	SMG1_L	occMed_R
A160	SMA2_L	IFG5_L
A168	SMA2_L	tempMed_L
A133	SMA1_L	SMG3_L
A141	SMA1_R	IFG4_L
